# 2-(4-Fluoro­phen­yl)-3-[5-(4-nitro­phen­yl)-1,3,4-thia­diazol-2-yl]-1,3-thia­zolidin-4-one

**DOI:** 10.1107/S1600536810023354

**Published:** 2010-06-23

**Authors:** Peng Yu, Kang An, Qiu He, Jian-Qaing Zhang, Rong Wan

**Affiliations:** a Department of Applied Chemistry, College of Science, Nanjing University of Technology, No. 5 Xinmofan Road, Nanjing, Nanjing 210009, People’s Republic of China

## Abstract

In the title compound, C_17_H_11_FN_4_O_3_S_2_, the five-membered thia­zolidinone and thia­diazole rings are almost planar, with r.m.s. deviations of 0.017 and 0.0019 Å, respectively. The 4-fluoro­phenyl ring is almost perpendicular to the thia­diazole ring, making a dihedral angle of 89.5 (3)°. The 4-nitro­phenyl ring is nearly coplanar with the thia­diazole ring, the dihedral angle being 7.9 (3)°. The crystal structure is stabilized by two inter­molecular C—H⋯O hydrogen bonds.

## Related literature

For the chemical and pharmaceutical properties of thia­diazole derivatives, see: Arun *et al.* (1999 ▶); Chen *et al.* (2000 ▶); Kidwai *et al.* (2000 ▶); Vicentini *et al.* (1998 ▶); Wasfy *et al.* (1996 ▶). For a related structure, see: Wan *et al.* (2008 ▶). For bond-length data, see: Allen *et al.* (1987 ▶).
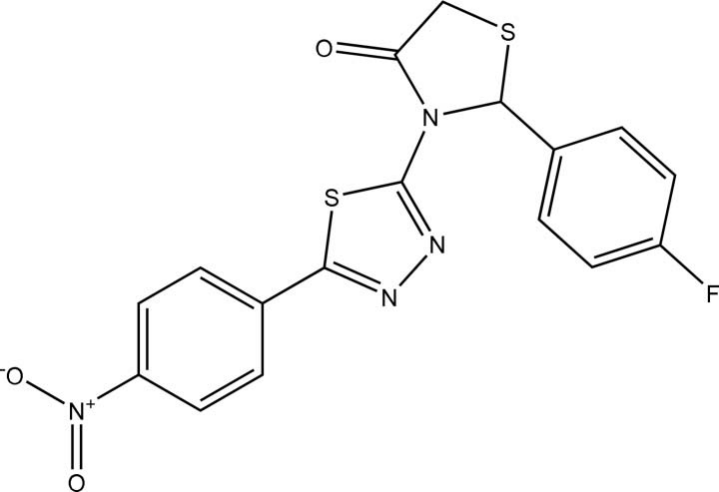


## Experimental

### 

#### Crystal data

C_17_H_11_FN_4_O_3_S_2_
*M* *_r_* = 402.44
Triclinic, 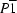

*a* = 7.2360 (14) Å
*b* = 9.1340 (18) Å
*c* = 14.464 (3) Å
α = 71.67 (2)°
β = 87.16 (3)°
γ = 75.69 (2)°
*V* = 878.9 (3) Å^3^
*Z* = 2
Mo *K*α radiation
μ = 0.34 mm^−1^
*T* = 293 K
0.30 × 0.10 × 0.05 mm

#### Data collection

Enraf–Nonius CAD-4 diffractometer
Absorption correction: ψ scan (North *et al.*, 1968 ▶) *T*_min_ = 0.905, *T*_max_ = 0.983
3470 measured reflections
3195 independent reflections
1330 reflections with *I* > 2σ(*I*)
*R*_int_ = 0.036
3 standard reflections every 200 reflections  intensity decay: 1%

#### Refinement

*R*[*F* ^2^ > 2σ(*F* ^2^)] = 0.060
*wR*(*F* ^2^) = 0.086
*S* = 0.96
3195 reflections
244 parameters
2 restraints
H-atom parameters constrained
Δρ_max_ = 0.24 e Å^−3^
Δρ_min_ = −0.16 e Å^−3^

### 

Data collection: *CAD-4 EXPRESS* (Enraf-Nonius, 1989 ▶); cell refinement: *CAD-4 EXPRESS*; data reduction: *XCAD4* (Harms & Wocadlo, 1995 ▶); program(s) used to solve structure: *SHELXS97* (Sheldrick, 2008 ▶); program(s) used to refine structure: *SHELXL97* (Sheldrick, 2008 ▶); molecular graphics: *SHELXTL* (Sheldrick, 2008 ▶); software used to prepare material for publication: *SHELXL97*.

## Supplementary Material

Crystal structure: contains datablocks global, I. DOI: 10.1107/S1600536810023354/pv2286sup1.cif

Structure factors: contains datablocks I. DOI: 10.1107/S1600536810023354/pv2286Isup2.hkl

Additional supplementary materials:  crystallographic information; 3D view; checkCIF report

## Figures and Tables

**Table 1:** Hydrogen-bond geometry (Å, °)

*D*—H⋯*A*	*D*—H	H⋯*A*	*D*⋯*A*	*D*—H⋯*A*
C5—H5*A*⋯O2^i^	0.93	2.56	3.411 (7)	152
C14—H14*A*⋯O1^ii^	0.93	2.52	3.198 (6)	130

Symmetry codes: (i) 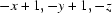; (ii) 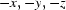.

**Fig. 1.**
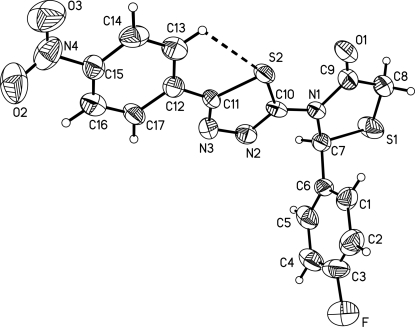
A view of the molecular structure of (I). Displacement ellipsoids are drawn at the 50% probability level. Dashed lines indicate intramolecular C—H···S hydrogen bond.

**Fig. 2.**
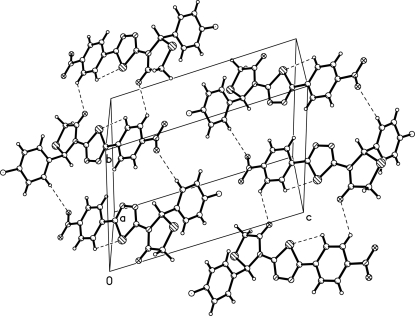
A packing diagram for (I). Dashed lines indicate intramolecular C—H···S hydrogen bond, and intermolecular C—H···O hydrogen bonds.

## supplementary crystallographic information

### Comment

1,3,4-Thiadiazole derivatives containing thiazolidinone unit are of great interest because of their chemical and pharmaceutical properties. Some derivatives have fungicidal activities and exhibit herbicidal activities (Chen *et al.*, 2000; Kidwai *et al.*, 2000; Vicentini *et al.*, 1998). Some thiadiazole derivatives show insecticidal activities (Arun *et al.*, 1999; Wasfy *et al.*,1996). We report here the crystal structure of the titled compound,(I).

The molecular strucutre of (I) is shown in Fig. 1. The bond distances and bond angles in (I) are normal (Allen *et al.*, 1987). In the title structure, unlike the structure of a related compound reported previously (Wan *et al.*, 2008), ring A (C7/S1/C8/C9/N1) is a planar five-mermbered ring and the mean deviation from the plane is 0.0170 Å. Rings B (C1—C6), C (S2/C10/N2/N3/C11) and D (C12—C17) are also individually planar. The dihedral angles between the mean-planes of the rings are: A/B = 85.2 (2) °, A/C = 7.3 (3) °, A/D = 1.1 (1) °, B/C = 89.5 (2) °, B/D = 85.6 (3)° and C/D = 7.9 (3) °. The intramolecular C—H···S hydrogen bond (Tab. 1) results in the formation of a planar five-membered ring (S2/C11/C12/C13/H13A). In the crystal structure, intermolecular C—H···O hydrogen bonds link the molecules to form a dimeric unit (Tab. 1 & Fig. 2), which may be effective in the stabilization of the structure.

### Experimental

*N*-(4-Fluorobenzylidene)-5-(4-nitrophenyl)-1,3,4-thiadiazol-2-amine and mercapto-acetic acid (5 mmol) were added in toluene (50 ml). The water produced in the reaction was collected in a Dean-Stark water separator. The reaction mixture was left to cool to room temperature, filtered, and the filterate was crystallized from acetone to give pure compound (I) (m.p. 468–469 K). Crystals of (I) suitable for X-ray diffraction were obtained by slow evaporation of an acetone solution.

### Refinement

All H atoms were positioned geometrically, with C—H = 0.98, 0.97, 0.96 and 0.93 Å for methine, methylene, methyl and aromatic H atoms, respectively, and constrained to ride on their parent atoms, with *U*_iso_(H) = *xU*_eq_(C), where *x* = 1.5 for methyl H atoms and *x* = 1.2 for all other H atoms.

### Crystal data

**Table:**

C_17_H_11_FN_4_O_3_S_2_	*Z* = 2
*M**_r_* = 402.44	*F*(000) = 412
Triclinic, *P*1	*D*_x_ = 1.521 Mg m^−^^3^
Hall symbol: -P 1	Melting point = 468–469 K
*a* = 7.2360 (14) Å	Mo *K*α radiation, λ = 0.71073 Å
*b* = 9.1340 (18) Å	Cell parameters from 25 reflections
*c* = 14.464 (3) Å	θ = 8–12°
α = 71.67 (2)°	µ = 0.34 mm^−^^1^
β = 87.16 (3)°	*T* = 293 K
γ = 75.69 (2)°	Plate, colorless
*V* = 878.9 (3) Å^3^	0.30 × 0.10 × 0.05 mm

### Data collection

**Table:**

Enraf–Nonius CAD-4 diffractometer	1330 reflections with *I* > 2σ(*I*)
Radiation source: fine-focus sealed tube	*R*_int_ = 0.036
graphite	θ_max_ = 25.3°, θ_min_ = 1.5°
ω/2θ scans	*h* = 0→8
Absorption correction: ψ scan (North *et al.*, 1968)	*k* = −10→10
*T*_min_ = 0.905, *T*_max_ = 0.983	*l* = −17→17
3470 measured reflections	3 standard reflections every 200 reflections
3195 independent reflections	intensity decay: 1%

### Refinement

**Table:**

Refinement on *F*^2^	Primary atom site location: structure-invariant direct methods
Least-squares matrix: full	Secondary atom site location: difference Fourier map
*R*[*F*^2^ > 2σ(*F*^2^)] = 0.060	Hydrogen site location: inferred from neighbouring sites
*wR*(*F*^2^) = 0.086	H-atom parameters constrained
*S* = 0.96	*w* = 1/[σ^2^(*F*_o_^2^) + (0.010*P*)^2^] where *P* = (*F*_o_^2^ + 2*F*_c_^2^)/3
3195 reflections	(Δ/σ)_max_ < 0.001
244 parameters	Δρ_max_ = 0.24 e Å^−^^3^
2 restraints	Δρ_min_ = −0.16 e Å^−^^3^

### Special details

**Table:**

Geometry. All e.s.d.'s (except the e.s.d. in the dihedral angle between two l.s. planes) are estimated using the full covariance matrix. The cell e.s.d.'s are taken into account individually in the estimation of e.s.d.'s in distances, angles and torsion angles; correlations between e.s.d.'s in cell parameters are only used when they are defined by crystal symmetry. An approximate (isotropic) treatment of cell e.s.d.'s is used for estimating e.s.d.'s involving l.s. planes.
Refinement. Refinement of *F*^2^ against ALL reflections. The weighted *R*-factor *wR* and goodness of fit *S* are based on *F*^2^, conventional *R*-factors *R* are based on *F*, with *F* set to zero for negative *F*^2^. The threshold expression of *F*^2^ > σ(*F*^2^) is used only for calculating *R*-factors(gt) *etc*. and is not relevant to the choice of reflections for refinement. *R*-factors based on *F*^2^ are statistically about twice as large as those based on *F*, and *R*- factors based on ALL data will be even larger.

### Fractional atomic coordinates and isotropic or equivalent isotropic displacement parameters (Å^2^)

**Table:**

	*x*	*y*	*z*	*U*_iso_*/*U*_eq_	
S1	−0.38553 (16)	0.40768 (16)	0.33761 (10)	0.1076 (5)	
S2	0.04152 (14)	0.21307 (12)	0.06830 (8)	0.0692 (3)	
F	0.4006 (4)	0.1694 (3)	0.5770 (2)	0.1367 (11)	
O1	−0.2824 (4)	0.1676 (4)	0.1585 (2)	0.0930 (11)	
O2	0.9132 (6)	0.1639 (5)	−0.2536 (3)	0.1480 (18)	
O3	0.7277 (6)	0.0551 (5)	−0.2946 (3)	0.1517 (18)	
N1	−0.1477 (4)	0.3266 (4)	0.2108 (2)	0.0661 (9)	
N2	0.1157 (5)	0.4161 (3)	0.1383 (2)	0.0683 (10)	
N3	0.2535 (5)	0.3935 (4)	0.0692 (2)	0.0762 (11)	
N4	0.7610 (8)	0.1341 (6)	−0.2506 (4)	0.1108 (18)	
C1	0.0664 (6)	0.1986 (6)	0.3988 (3)	0.0880 (15)	
H1A	0.0117	0.1288	0.3813	0.106*	
C2	0.2070 (8)	0.1378 (7)	0.4716 (4)	0.1105 (19)	
H2B	0.2581	0.0290	0.4985	0.133*	
C3	0.2620 (7)	0.2377 (9)	0.4996 (4)	0.105 (2)	
C4	0.2190 (8)	0.4000 (8)	0.4583 (4)	0.1027 (19)	
H4A	0.2763	0.4664	0.4780	0.123*	
C5	0.0798 (7)	0.4547 (6)	0.3836 (4)	0.0941 (16)	
H5A	0.0356	0.5637	0.3538	0.113*	
C6	0.0073 (6)	0.3531 (6)	0.3532 (3)	0.0699 (12)	
C7	−0.1554 (5)	0.4201 (4)	0.2769 (3)	0.0673 (12)	
H7A	−0.1582	0.5308	0.2398	0.081*	
C8	−0.4389 (6)	0.2787 (5)	0.2801 (3)	0.0997 (14)	
H8A	−0.4480	0.1799	0.3289	0.120*	
H8B	−0.5612	0.3270	0.2454	0.120*	
C9	−0.2846 (7)	0.2443 (6)	0.2088 (4)	0.0880 (16)	
C10	−0.0012 (5)	0.3287 (4)	0.1446 (3)	0.0618 (11)	
C11	0.2339 (6)	0.2965 (5)	0.0283 (3)	0.0618 (12)	
C12	0.3617 (6)	0.2569 (5)	−0.0445 (3)	0.0707 (12)	
C13	0.3348 (6)	0.1556 (5)	−0.0957 (3)	0.0773 (13)	
H13A	0.2304	0.1107	−0.0828	0.093*	
C14	0.4619 (7)	0.1221 (5)	−0.1651 (3)	0.0899 (15)	
H14A	0.4381	0.0628	−0.2029	0.108*	
C15	0.6262 (7)	0.1787 (5)	−0.1773 (3)	0.0748 (13)	
C16	0.6601 (6)	0.2724 (6)	−0.1301 (3)	0.0924 (15)	
H16A	0.7680	0.3127	−0.1423	0.111*	
C17	0.5285 (6)	0.3094 (5)	−0.0611 (3)	0.0749 (13)	
H17A	0.5540	0.3712	−0.0254	0.090*	

### Atomic displacement parameters (Å^2^)

**Table:**

	*U*^11^	*U*^22^	*U*^33^	*U*^12^	*U*^13^	*U*^23^
S1	0.0478 (7)	0.1601 (13)	0.1320 (12)	−0.0182 (8)	0.0108 (8)	−0.0759 (11)
S2	0.0633 (7)	0.0719 (8)	0.0791 (8)	−0.0314 (6)	−0.0064 (6)	−0.0202 (6)
F	0.130 (3)	0.152 (3)	0.127 (2)	−0.024 (2)	−0.024 (2)	−0.047 (2)
O1	0.080 (2)	0.109 (3)	0.109 (3)	−0.049 (2)	0.001 (2)	−0.040 (2)
O2	0.150 (4)	0.155 (4)	0.172 (4)	−0.060 (3)	0.080 (4)	−0.090 (3)
O3	0.139 (4)	0.198 (4)	0.144 (4)	−0.012 (3)	0.007 (3)	−0.113 (3)
N1	0.051 (2)	0.087 (3)	0.063 (2)	−0.024 (2)	−0.0025 (19)	−0.021 (2)
N2	0.069 (2)	0.061 (2)	0.078 (3)	−0.015 (2)	−0.004 (2)	−0.027 (2)
N3	0.074 (3)	0.089 (3)	0.072 (3)	−0.035 (2)	0.010 (2)	−0.025 (2)
N4	0.112 (4)	0.113 (4)	0.083 (4)	0.012 (4)	0.017 (4)	−0.029 (3)
C1	0.069 (3)	0.091 (4)	0.076 (4)	−0.004 (3)	−0.002 (3)	0.002 (3)
C2	0.099 (5)	0.114 (5)	0.092 (5)	−0.009 (4)	0.001 (3)	−0.008 (4)
C3	0.066 (4)	0.160 (7)	0.091 (5)	−0.011 (5)	−0.013 (3)	−0.054 (5)
C4	0.077 (4)	0.151 (5)	0.119 (5)	−0.045 (4)	0.019 (4)	−0.085 (5)
C5	0.066 (3)	0.126 (5)	0.111 (4)	−0.028 (3)	0.027 (3)	−0.065 (4)
C6	0.047 (3)	0.106 (4)	0.058 (3)	−0.020 (3)	0.004 (2)	−0.028 (3)
C7	0.063 (3)	0.079 (3)	0.068 (3)	−0.023 (3)	0.004 (2)	−0.029 (3)
C8	0.076 (3)	0.146 (4)	0.084 (4)	−0.051 (3)	0.008 (3)	−0.027 (3)
C9	0.070 (3)	0.110 (5)	0.080 (4)	−0.037 (3)	−0.004 (3)	−0.011 (3)
C10	0.046 (3)	0.068 (3)	0.073 (3)	−0.013 (2)	−0.013 (2)	−0.024 (3)
C11	0.072 (3)	0.066 (3)	0.061 (3)	−0.043 (2)	0.004 (2)	−0.020 (2)
C12	0.076 (3)	0.067 (3)	0.066 (3)	−0.025 (3)	−0.006 (3)	−0.009 (3)
C13	0.081 (3)	0.064 (3)	0.093 (4)	−0.017 (3)	−0.003 (3)	−0.032 (3)
C14	0.120 (5)	0.079 (3)	0.082 (4)	−0.030 (3)	−0.020 (3)	−0.032 (3)
C15	0.079 (4)	0.066 (3)	0.071 (4)	−0.027 (3)	−0.001 (3)	−0.002 (3)
C16	0.073 (4)	0.111 (4)	0.096 (4)	−0.024 (3)	−0.008 (3)	−0.033 (3)
C17	0.066 (3)	0.081 (3)	0.087 (4)	−0.036 (3)	−0.002 (3)	−0.025 (3)

### Geometric parameters (Å, °)

**Table:**

S1—C8	1.761 (4)	C4—C5	1.397 (6)
S1—C7	1.855 (3)	C4—H4A	0.9300
S2—C10	1.723 (4)	C5—C6	1.360 (5)
S2—C11	1.738 (3)	C5—H5A	0.9300
F—C3	1.418 (5)	C6—C7	1.526 (5)
O1—C9	1.155 (5)	C7—H7A	0.9800
O2—N4	1.194 (5)	C8—C9	1.525 (5)
O3—N4	1.172 (5)	C8—H8A	0.9700
N1—C9	1.390 (5)	C8—H8B	0.9700
N1—C10	1.392 (4)	C11—C12	1.439 (5)
N1—C7	1.460 (4)	C12—C17	1.389 (5)
N2—C10	1.281 (4)	C12—C13	1.406 (4)
N2—N3	1.401 (4)	C13—C14	1.381 (5)
N3—C11	1.248 (4)	C13—H13A	0.9300
N4—C15	1.489 (6)	C14—C15	1.391 (5)
C1—C6	1.324 (5)	C14—H14A	0.9300
C1—C2	1.382 (6)	C15—C16	1.321 (5)
C1—H1A	0.9300	C16—C17	1.405 (5)
C2—C3	1.257 (6)	C16—H16A	0.9300
C2—H2B	0.9300	C17—H17A	0.9300
C3—C4	1.373 (6)		
			
C8—S1—C7	95.25 (18)	C9—C8—S1	110.5 (3)
C10—S2—C11	86.21 (19)	C9—C8—H8A	109.6
C9—N1—C10	120.0 (4)	S1—C8—H8A	109.6
C9—N1—C7	122.6 (4)	C9—C8—H8B	109.6
C10—N1—C7	117.3 (3)	S1—C8—H8B	109.6
C10—N2—N3	110.6 (3)	H8A—C8—H8B	108.1
C11—N3—N2	114.2 (3)	O1—C9—N1	124.8 (5)
O3—N4—O2	121.2 (6)	O1—C9—C8	126.7 (5)
O3—N4—C15	120.6 (6)	N1—C9—C8	108.5 (4)
O2—N4—C15	117.5 (6)	N2—C10—N1	121.3 (4)
C6—C1—C2	122.1 (5)	N2—C10—S2	115.0 (3)
C6—C1—H1A	118.9	N1—C10—S2	123.6 (3)
C2—C1—H1A	118.9	N3—C11—C12	122.5 (4)
C3—C2—C1	116.3 (6)	N3—C11—S2	113.9 (3)
C3—C2—H2B	121.8	C12—C11—S2	123.6 (3)
C1—C2—H2B	121.8	C17—C12—C13	116.9 (4)
C2—C3—C4	128.0 (6)	C17—C12—C11	119.8 (4)
C2—C3—F	114.2 (7)	C13—C12—C11	123.2 (4)
C4—C3—F	117.4 (6)	C14—C13—C12	120.7 (4)
C3—C4—C5	112.7 (5)	C14—C13—H13A	119.7
C3—C4—H4A	123.7	C12—C13—H13A	119.7
C5—C4—H4A	123.7	C13—C14—C15	118.8 (4)
C6—C5—C4	121.9 (5)	C13—C14—H14A	120.6
C6—C5—H5A	119.1	C15—C14—H14A	120.6
C4—C5—H5A	119.1	C16—C15—C14	122.9 (5)
C1—C6—C5	118.4 (5)	C16—C15—N4	120.7 (5)
C1—C6—C7	121.8 (5)	C14—C15—N4	116.4 (5)
C5—C6—C7	119.4 (5)	C15—C16—C17	118.2 (5)
N1—C7—C6	113.5 (3)	C15—C16—H16A	120.9
N1—C7—S1	102.9 (2)	C17—C16—H16A	120.9
C6—C7—S1	109.6 (3)	C12—C17—C16	122.3 (4)
N1—C7—H7A	110.2	C12—C17—H17A	118.9
C6—C7—H7A	110.2	C16—C17—H17A	118.9
S1—C7—H7A	110.2		
			
C10—N2—N3—C11	0.7 (5)	N3—N2—C10—S2	−0.5 (4)
C6—C1—C2—C3	7.2 (8)	C9—N1—C10—N2	173.0 (4)
C1—C2—C3—C4	−9.5 (9)	C7—N1—C10—N2	−3.8 (5)
C1—C2—C3—F	177.5 (4)	C9—N1—C10—S2	−8.7 (5)
C2—C3—C4—C5	7.9 (9)	C7—N1—C10—S2	174.5 (3)
F—C3—C4—C5	−179.4 (4)	C11—S2—C10—N2	0.2 (3)
C3—C4—C5—C6	−3.8 (7)	C11—S2—C10—N1	−178.2 (3)
C2—C1—C6—C5	−4.0 (7)	N2—N3—C11—C12	−179.7 (3)
C2—C1—C6—C7	−176.6 (4)	N2—N3—C11—S2	−0.6 (5)
C4—C5—C6—C1	2.5 (6)	C10—S2—C11—N3	0.2 (3)
C4—C5—C6—C7	175.2 (4)	C10—S2—C11—C12	179.4 (4)
C9—N1—C7—C6	113.8 (4)	N3—C11—C12—C17	9.3 (6)
C10—N1—C7—C6	−69.5 (4)	S2—C11—C12—C17	−169.8 (3)
C9—N1—C7—S1	−4.6 (4)	N3—C11—C12—C13	−175.9 (4)
C10—N1—C7—S1	172.1 (3)	S2—C11—C12—C13	5.0 (6)
C1—C6—C7—N1	−41.7 (5)	C17—C12—C13—C14	−5.3 (6)
C5—C6—C7—N1	145.9 (4)	C11—C12—C13—C14	179.8 (4)
C1—C6—C7—S1	72.8 (5)	C12—C13—C14—C15	5.7 (7)
C5—C6—C7—S1	−99.7 (4)	C13—C14—C15—C16	−4.8 (7)
C8—S1—C7—N1	3.4 (3)	C13—C14—C15—N4	177.9 (4)
C8—S1—C7—C6	−117.7 (3)	O3—N4—C15—C16	−177.6 (5)
C7—S1—C8—C9	−2.0 (3)	O2—N4—C15—C16	11.9 (8)
C10—N1—C9—O1	4.3 (7)	O3—N4—C15—C14	−0.2 (7)
C7—N1—C9—O1	−179.1 (5)	O2—N4—C15—C14	−170.8 (5)
C10—N1—C9—C8	−173.2 (3)	C14—C15—C16—C17	3.3 (7)
C7—N1—C9—C8	3.4 (5)	N4—C15—C16—C17	−179.5 (4)
S1—C8—C9—O1	−177.7 (5)	C13—C12—C17—C16	3.9 (6)
S1—C8—C9—N1	−0.2 (5)	C11—C12—C17—C16	179.0 (4)
N3—N2—C10—N1	177.9 (3)	C15—C16—C17—C12	−2.9 (7)

### Hydrogen-bond geometry (Å, °)

**Table:**

*D*—H···*A*	*D*—H	H···*A*	*D*···*A*	*D*—H···*A*
C5—H5A···O2^i^	0.93	2.56	3.411 (7)	152
C13—H13A···S2	0.93	2.81	3.184 (5)	106
C14—H14A···O1^ii^	0.93	2.52	3.198 (6)	130

Symmetry codes: (i) −*x*+1, −*y*+1, −*z*; (ii) −*x*, −*y*, −*z*.
